# Α 10-gigawatt attosecond source for non-linear XUV optics and XUV-pump-XUV-probe studies

**DOI:** 10.1038/s41598-020-60331-9

**Published:** 2020-02-28

**Authors:** I. Makos, I. Orfanos, A. Nayak, J. Peschel, B. Major, I. Liontos, E. Skantzakis, N. Papadakis, C. Kalpouzos, M. Dumergue, S. Kühn, K. Varju, P. Johnsson, A. L’Huillier, P. Tzallas, D. Charalambidis

**Affiliations:** 1 Foundation for Research and Technology - Hellas, Institute of Electronic Structure & Laser, GR71110 Heraklion, Crete Greece; 2 Department of Physics, University of Crete, GR71003 Heraklion, Crete Greece; 3 ELI-ALPS, ELI-Hu Non-Profit Ltd., Dugonics tér 13, H-6720 Szeged, Hungary; 4 Department of Optics and Quantum Electronics, University of Szeged, Dom tér 9, 6720 Szeged, Hungary; 5 Department of Physics, Lund University, SE-221 00 Lund, Sweden; 6 Institute of Physics, University of Szeged, Dom tér 9, 6720 Szeged, Hungary

**Keywords:** Atomic and molecular interactions with photons, Attosecond science

## Abstract

The quantum mechanical motion of electrons and nuclei in systems spatially confined to the molecular dimensions occurs on the sub-femtosecond to the femtosecond timescales respectively. Consequently, the study of ultrafast electronic and, in specific cases, nuclear dynamics requires the availability of light pulses with attosecond (asec) duration and of sufficient intensity to induce two-photon processes, essential for probing the intrinsic system dynamics. The majority of atoms, molecules and solids absorb in the extreme-ultraviolet (XUV) spectral region, in which the synthesis of the required attosecond pulses is feasible. Therefore, the XUV spectral region optimally serves the study of such ultrafast phenomena. Here, we present a detailed review of the first 10-GW class XUV attosecond source based on laser driven high harmonic generation in rare gases. The pulse energy of this source largely exceeds other laser driven attosecond sources and is comparable to the pulse energy of femtosecond Free-Electron-Laser (FEL) XUV sources. The measured pulse duration in the attosecond pulse train is 650 ± 80 asec. The uniqueness of the combined high intensity and short pulse duration of the source is evidenced in non-linear XUV-optics experiments. It further advances the implementation of XUV-pump-XUV-probe experiments and enables the investigation of strong field effects in the XUV spectral region.

## Introduction

In the 20 years of attosecond science^1,2^, numerous exciting ideas have been conceived and sound applications have been demonstrated, the majority of which is based on pump-probe studies, exploiting combinations of infrared (IR) and XUV pulses.

Already the domain of attosecond pulse characterization gave access to fascinating physics, novel methodologies and innovative technologies. Those are to be found in the Reconstruction of Attosecond Beating By Interference of two-photon Transitions (RABBIT)^3^, Frequency Resolved Optical Gating for Complete Reconstruction of Attosecond Bursts (FROG-CRAB)^4^, Phase Retrieval by Omega Oscillation Filtering (PROOF)^5,6^, Rainbow RABBIT^7^, *In-situ*^8^, Spectral Phase Interferometry for the Direct Electric Field Reconstruction (SPIDER)^9,10^, atto-clock^11^, double-blind holography^12^, attosecond spatial interferometry^13^, and the attosecond streaking^14^ methods and in the devices developed towards their implementation. A summary of these approaches is presented in the perspective article on attosecond pulse metrology^15^.

In parallel, abundant, significant proof of principle experiments enriched the pallet of attosecond applications. Atomic inner-shell spectroscopy^16^, real-time observation of ionization^17^, light wave electronics^18^, and molecular optical tomography^19^ are some examples of such experiments. Other more recent applications of attosecond pulses include ionization delays in solids^20^ and atoms^21,22^, electron dynamics^23^, charge migration^24,25^, build-up of a Fano-Beutler resonance^7^, and ionization dynamics in chiral molecules^26^. It should be noted that the above examples are only a representative fraction of many studies performed in attosecond laboratories.

Following a somewhat different path, a group of attosecond laboratories focused for several years their efforts towards the development of high photon flux attosecond beam lines. The aim of these efforts was to reach sufficiently high attosecond pulse intensities as to induce observable two- (or more) XUV-photon transitions, a central prerequisite for XUV-pump-XUV-probe experiments in the one femtosecond (fs) and sub-fs temporal regime^27–29^. The importance of XUV-pump-XUV-probe schemes relies on the fact that when temporarily overlapping IR and XUV pulses are used for pump-probe studies, the high IR intensities that have to be employed may cause distortions to the system under investigation obscuring its intrinsic dynamics^30^. XUV-pump-XUV-probe experiments benefit substantially from the existence of intense isolated^27,28^ or essentially isolated^31^ XUV pulses. At the same time, observable two-(or more) XUV-photon transitions allow temporal characterization of attosecond pulses based on non-linear XUV autocorrelation (AC) measurements^32–38^, bypassing complications that may arise from IR-XUV cross-correlation based pulse characterization techniques^39^. It should be noted that these developments were a follow up of pioneering non-linear XUV experiments completed with individual harmonics in the few tens of fs temporal regime, including two-^40^, three-^41^ and four-XUV-photon^42^ ionization, two-XUV-photon double ionization^43,44^ as well as the corresponding 2^nd^ ^40,43^ and 4^th^ order AC measurements^42^, two-XUV-photon above threshold ionization (ATI)^45^ and even a FROG based XUV pulse reconstruction^46^.

Towards reaching high XUV photon fluxes there are certain hurdles including depletion of the generating medium above a certain threshold of the driving laser intensity, XUV radiation reabsorption by the generating medium, as well as phase mismatch due to high generating gas pressures and high degree of ionization of the generating medium (see the review article of ref. ^47^). A way to overcome these obstacles is to use non depleting media as non-linear harmonic generation targets. This is the case in the generation of harmonics from laser induced surface plasma^48–53^, often referred to as plasma mirrors^54^. Indeed, for surface plasma harmonics, very high photon fluxes have been predicted in particle in cell (PIC) simulations^55^ and sub-fs temporal confinement has been experimentally demonstrated^56^. Laser surface plasma harmonic generation requires however, increased technological demands such as high laser peak to background contrast, including elimination of unwanted laser pre-pulses, demanding “cleaning” procedures of the laser pulse through additional plasma mirrors, tedious control of the plasma density gradient^53^, μm positioning of the focus on the target and debris to mention a few. Although laser surface plasma harmonic generation holds promise of high photon flux attosecond pulses, the so far achieved maximum XUV pulse energy is 40 μJ^56^.

The alternative to laser surface plasma harmonic generation in avoiding the above mentioned obstacles is to use gas targets combined with loose focusing of the driving laser beam. The scalability of gas phase harmonic generation sources has been recently studied in ref. ^57^. The work by Heyl *et al*. demonstrates that long focal lengths combined with low pressure gas cells, allowing control of phase matching, can lead to high throughputs and thus to high XUV photon fluxes. At the same time it has been recently shown that multi-cycle high peak power laser beams, focused in the generation medium using long focal lengths of several meters, in combination with quasi-phase matching^58^ arrangements, achieved through a chain of small length gas media i.e. pulsed gas jets, can reach emission of 20-GW XUV harmonic power at the source in the spectral region of 15–30 eV^59^. In the work of Nayak *et al*. apart from the measurement of the harmonic source power the high focused XUV intensities achieved were evidenced through the observation of multi-XUV-photon multiple ionization of argon atoms. While FEL sources have much higher peak brightness at shorter wavelengths and in particular in the x-ray regime, in the spectral region of 15–30 eV the measured peak brightness of the harmonic spectra is competing with that of FELs^52^.

In the present work we provide an in-detail presentation of the 20-GW XUV source developed at the Instutute of Electronic Structure and Laser of the Foundation for Research and Technology-Hellas (FORTH-IESL) together with multi-XUV-photon multiple atomic ionization measurements in helium, argon and neon, while 10 GW attosecond pulse trains have been demonstrated at this source. Two-photon ionization of helium atoms and argon ions is used in second order intensity volume autocorrelation (2^nd^ IVAC) measurements of the pulse duration of the attosecond pulse train (APT). Since the measured duration of the pulses in the train is found to be τ_XUV_ = 670 ± 80 asec and τ_XUV_ = 650 ± 80 asec in He and Ar respectively, the present work introduces the most powerful table top XUV attosecond source.

The structure of the manuscript is as follows. In section 2 we give a detailed illustration of the XUV beam-line. In section 3 we report a quantitative characterization of the different parameters of the beam-line. In section 4 we present results of non-linear XUV-optics experiments. In section 5 results of the attosecond pulse trains temporal characterization are shown, followed by the concluding section of the work. It should be noted that after submission of the present work tunable attosecond x-ray pulses with 100 GW peak power were demonstrated in the SLAC FEL large scale infrastructure^60^.

## The High XUV Photon Flux Source

The high XUV-photon flux beam-line mentioned in the previous section has been recently developed and tested in the Attosecond Science & Technology laboratory of FORTH-IESL^59^. In this section, a detailed description of the beam line and its characterization is presented.

### The 20-GW XUV beam-line

The high photon throughput of the XUV beam-line relies on the exploitation of: I) 9 m focal length optics focusing the laser beam into the non-linear medium, as to increase the number of harmonic emitters in the interaction cross section, keeping the driving intensity below the ionization saturation thresholds of the generating medium, II) a dual gas jet as target with variable jet distance as to achieve optimal phase matching, III) optimized gas pressure in both jets, and IV) Xe gas as non-linear medium, the conversion efficiency of which is the highest of all rare gasses^61,62^ with the trade-off of the low cut-off photon energy. However, in test measurements Ar gas was also used as generating medium.

**Figure 1:**
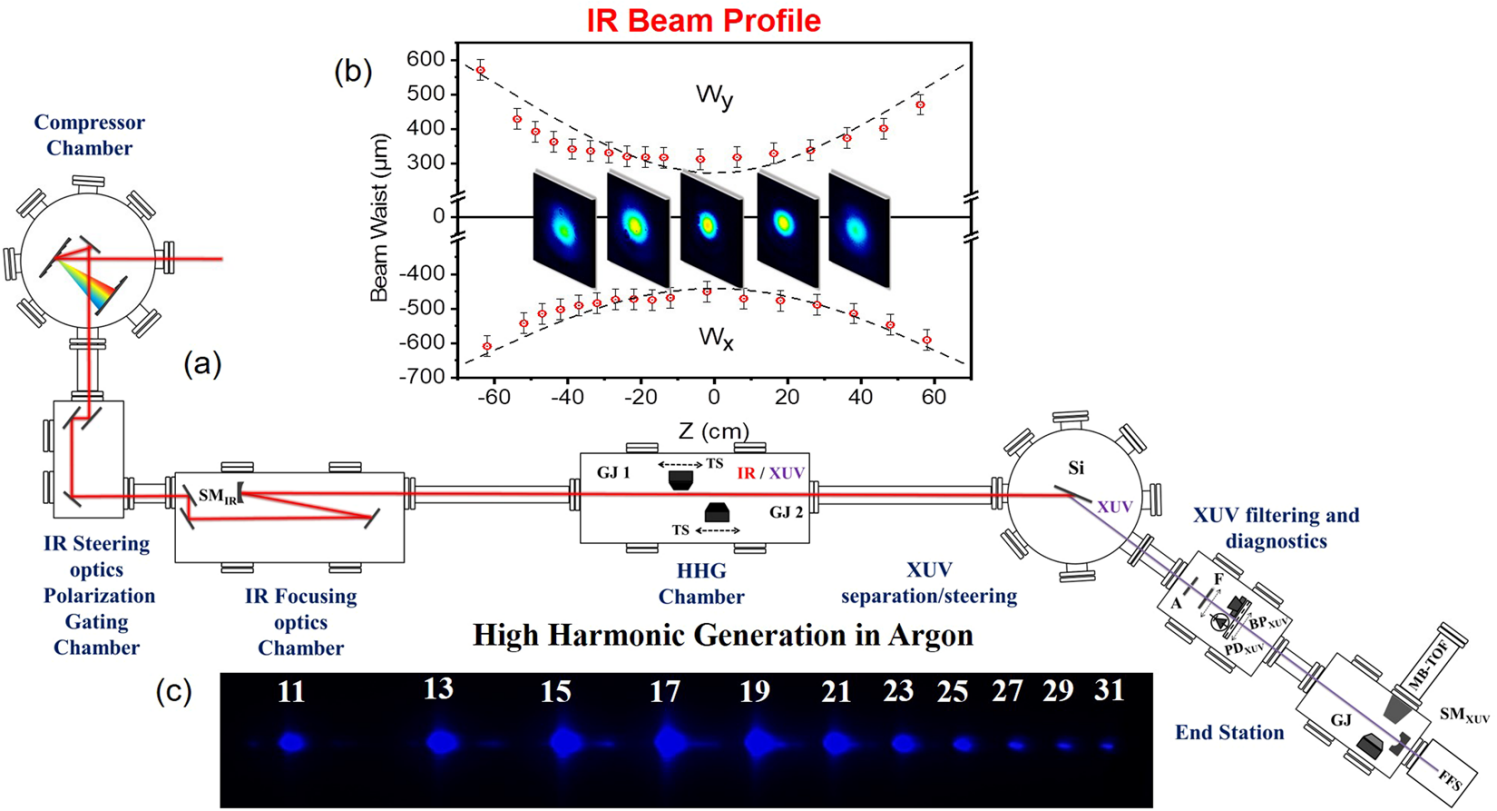
The 20-GW XUV beam line. (**a**) Optical layout of the 20-GW XUV beam-line. SM_IR_: spherical mirror of 9-m focal length. GJ_1,2_: dual-pulsed-jet configuration placed on translation stages (TS). Si: silicon plate. F: Al or Sn filter. A: aperture. BP_XUV_: XUV beam profiler. SM_XUV_: gold coated spherical mirror of 5-cm focal length. Ar-GJ: Ar gas jet. MB-TOF: magnetic bottle time-of-flight spectrometer. PD_XUV_: calibrated XUV photodiode. FFS: flat-field spectrometer. (**b**) IR beam profile around the focus measured with a CCD camera. (**c**) measured HHG spectrum produced in Argon gas phase medium spreading up to 48 eV corresponding to the 31^st^ harmonic of the fundamental frequency of the driving field. Part of the figure is copied from reference^59^.

The beam-line, as shown in Fig. 1, consists of the following units: (a) laser beam steering/shaping, placed in the “compressor chamber” and “IR steering optics and Polarization Gating” chambers, (b) laser beam focusing, placed in the “IR focusing optics” chamber, (c) XUV generation, placed in the “HHG” chamber, (d) XUV separation/steering, placed in the “XUV separation/steering” chamber, (e) XUV manipulation and diagnostics, placed in the “XUV filtering and diagnostics” chamber and (e) XUV pulse temporal characterization and XUV radiation use unit placed in the “end station”.

The laser steering and shaping takes place in two different vacuum chambers. In the first one a two grating arrangement compresses the amplified laser beam (Amplitude Technologies Ti:Sapphire chain) and delivers pulses of 800 nm central wavelength, ≈400 mJ maximum energy and ≈24 fs duration at 10 Hz repetition rate. Since 400 mJ pulse energy would deplete the harmonic generation medium at the used geometry the energy is reduced to 25–45 mJ after compression depending on the gas used for the generation.

The beam is then steered into the focusing unit through three plane mirrors placed in the second chamber. The same mirror set up is used for the alignment of the laser through the entire beam-line. This second chamber hosts also a Polarization Gating (PG) optical arrangement for the generation of isolated attosecond pulses. Since no isolated pulses are used in the present work the PG arrangement is not described here but can be found in previous works^63–65^. The polarization of the laser beam entering the focusing unit is *parallel* to the optical table. The beam diameter is *D* ≈ 2.3 cm. The focusing unit uses three silver protected low dispersion plane mirrors and a spherical mirror (SM) of 9 m focal length. The optical layout shown in Fig. 1a aims to reduce astigmatism introduced by the spherical mirror due to the deviation from the normal incidence. The angle of incidence at the spherical mirror is as close as possible to normal (~3°). In this way the astigmatism is kept low but is not negligible. Figure 1b shows the beam profile at the focus of the IR beam (measured with a CCD camera) which reveals a small degree of elongation along the x-axis. The confocal parameter is measured to be *b* ≈ 70 cm which is a factor of ≈ 1.22 larger than the value obtained according to the relation *b* = 2π*R*^2^/*λ*_L_ (where *R* and *λ*_L_ is the radius and the wavelength of the IR beam) given by Gaussian optics. Although these imperfections of the IR beam do not affect the XUV beam profile (measured with an XUV beam profiler placed after the metal filter in the “XUV diagnostics” chamber) as can be seen in Fig. 1 of ref. ^59^ and further down in this work, according to ref. ^66^, they may introduce distortions in XUV wavefront and hence influence the duration of the emitted attosecond pulses at the “end station” where the XUV beam is refocused. This matter will be further discussed in Section 4 of the manuscript. Further measurements of the IR profile have been performed at several positions around the focus as shown in Fig. 1b.

**Figure 2:**
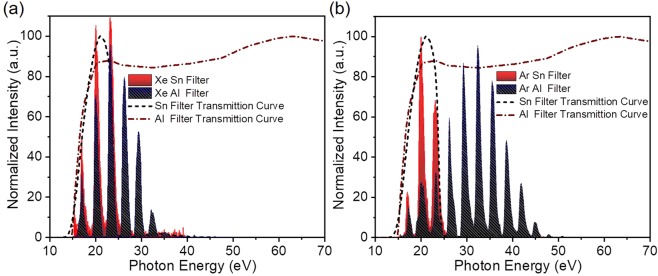
Harmonic spectra recorded by FFS after spectral selection by metallic foils. The generation medium was (**a**) Xe and (**b**) Ar gas. In both panels, the blue and red peaks correspond to harmonics after transmission through 150 nm thick Al and Sn respectively, while the red dash-dotted (Al) and black dashed (Sn) are transmission curves for 150 nm thickness.

The XUV generation unit can host up to four gas-jets placed on *x*, *y*, *z* translation stages. All gas-jets of the beamline are home made piezoelectric crystal based gas-jets. These translations are used for optimization of the laser-gas interaction. In addition, the translation in the *z* direction (beam propagation direction) permits the variation of the inter-jet distance, optimizing phase matching. Due to the large focal length, the distance between the jets is several cm and thus phase matching can be accurately controlled through translation in the *z* direction. The minimum step of the stage was 5 μm, much smaller than the needed accuracy in the range of cm. In the present study only two gas jets (GJ_1_, GJ_2_) have been used with the scanning step of the translation stages set at 0.75 cm. The gas jets are operated by piezoelectrically driven pulsed nozzles. For comparison reasons a 10 cm long gas cell bounded by two pinholes (entrance-exit pinholes) of 2 mm diameter has also been used in one of the experiments. The generated XUV co-propagates with the IR towards the “XUV separation/steering” chamber. The two beams (IR, XUV) first impinge a silicon plate (Si) placed at the Brewster angle (~75°) of the IR radiation. This plate significantly attenuates the IR and reflects ~60% of the XUV radiation deflecting the XUV beam towards the “XUV filtering and diagnostics” and “end station” chambers. In the “XUV filtering and diagnostics” chamber, the beam after passing through a 7 mm diameter aperture, is spectrally selected by 150 nm thick metal foils (Al or Sn) mounted on an *x*, *y* translation stage. The foils are acting as band pass filters in the XUV spectral range and eliminate any residual IR radiation. The transmission curve of these filters is shown in Fig. 2 together with harmonic spectra obtained using xenon (Fig. 2a) and argon (Fig. 2b) as generating gas, recorded by the XUV flat-field-spectrometer (FFS).

**Figure 3:**
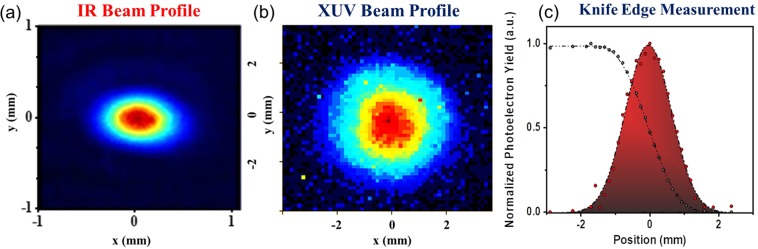
IR and XUV beam profiles. (**a**) IR beam profile at the focal plane measured by a commercial CCD profile camera. (**b**) XUV beam profile recorded using the BP_XUV_. For this measurement Xe gas was used as harmonic generation medium. (**c**) Knife edge measurement of the XUV beam profile presented with black dots while the red dots show the obtained intensity distribution. The colored area is defined by a Gaussian fit to the measured data. In both (**b**,**c**) measurements, harmonics are generated using xenon with the GJ placed at the IR focus.

In the “XUV filtering and diagnostics” chamber the pulse energy of the XUV radiation was also measured introducing a calibrated XUV photodiode (PD_XUV_) into the XUV beam and its beam profile was recorded introducing an XUV beam profiler (BP_XUV_) (consisting of a pair of multichannel plates (MCPs) and a phosphor screen followed by a CCD camera). Figure 3 shows the XUV beam profiles recorded after the filtering through Al foil with the GJ_1_ to be placed at the focusing position of the driving field. For further investigation, recordings have been carried out for several positions of the GJ_1_ producing the XUV radiation. No significant change was observed when GJ_1_ was placed before (z_GJ1_ = −b, −b/2), on (z_GJ1_ = 0) or after (z_GJ1_ = b, b/2) the driving laser focus. For an IR focus displacement of ≈±30 cm relative to the gas jet position, a significant change in the beam XUV profile is expected when both the short and long trajectory harmonics are recorded by the beam profiler. This is because, as it is well known, the divergence of the short trajectory harmonics is smaller than the long trajectory harmonics which have an annular-like beam profile. Focusing the IR beam before (after) the gas jet, the contribution of the short (long) trajectory harmonics is dominating. In the present measurements, the diameter of the aperture that has been placed before the beam profiler was reduced to ≈5 mm, thus selecting mainly the short trajectory harmonics (without excluding the presence of the long trajectories for harmonics lying close to the cut-off spectral region), and thus it does not “significantly” change when moving the jet before and after the focus. To double check the spatial intensity distribution of the XUV beam recorded by the BP_XUV_, the knife edge technique was also used for z_GJ1_ = 0. The XUV radiation photoionizes argon gas and the photoelectron yield is measured as a function of the knife edge position. The measured curve shown in Fig. 3c (black dots) is then differentiated resulting in the intensity distribution (red dots). The colored area is defined by a Gaussian fit to the measured data. The results of the knife edge measurements were in agreement with the values of the XUV beam radius obtained by the BP_XUV_.

The last chamber (end station) of the beam-line is the temporal characterization and pump-probe unit. It hosts an attosecond delay line based on a split spherical gold coated mirror of 5 cm focal length, fixed on a multiple-translation-rotation stage. This stage enables control in 3 degrees of freedom for the one D-shaped half of the mirror i.e. the displacement along the *z* axis (i.e. the beam propagation axis) with a maximum value of 80 μm and rotation in the x-z and y-z plane. The other part of the mirror position is altered only along the propagation direction with a maximum translation of 400 μm. All movements of the split-mirror are controlled by piezo crystals operated in closed loop mode. A 1.5 nm minimum step of the translation of the first, as described above, of the two parts of the bisected mirror introduces a temporal delay between the two parts of the beam. It is worth noting that for such time delays (80 μm total translation), effects of spatial displacements of the two parts of the focused beam are negligibly small^36^. The XUV beam is focused in front of a pulsed gas jet whose forefront serves also as a repeller of a magnetic bottle time of flight (MB-TOF) spectrometer. The TOF can be operated either in an ion mass spectrometer or electron energy analyzer mode measuring the products of the interaction of the XUV pulses with the gas target. This arrangement is used either for performing 2^nd^ IVAC measurements of the XUV pulse duration or in XUV-pump-XUV-probe experiments. Finally, the FFS is placed at the end of the beam line monitoring and recording the XUV radiation spectrum that is “leaking” through the slit of the bisected mirror.

## Characterization of the XUV Beam-line

In this section, vacuum, XUV pulse energy, attosecond delay line stability and temporal resolution measurements are discussed.

### Vacuum conditions

The rest vacuum, i.e. the vacuum when all gas jets are off, in all chambers of the beam-line is: ~10^−6^ mbar except for the “end station” chamber in which it is ~10^−7^ mbar. The generating nozzles are operating with a backing pressure in the range of 2 bar. The estimated gas pressure of the jets in the interaction area is ~25 mbar as reported in a previous work^59^. When the two generation jets are on, the pressure in the HHG chamber increases to ~10^−4^ mbar. The jet pressure conditions in the detection chamber depend on the type of experiment that is performed. A 1000 l/min turbo-molecular pump in the “end station” chamber secures an adequate vacuum pressure during operation of the gas target jet. An additional turbo pump differentially pumping the FFS spectrometer ensures that the pressure where the multichannel plate detector is located, is lower than 10^−6^ mbar.

### Measurement and optimization of the XUV pulse energy

Typical harmonic spectra generated in Ar and Xe, recorded by the FFS after the XUV radiation has passed through 150 nm thick Al or Sn filters are shown in Fig. 2. The harmonic cut-off region when Xe gas and Al filter are used is around 30 eV (and the highest harmonic observed is the 23^rd^), while harmonics higher than the 15^th^ are not transmitted through the Sn filter. In Ar the cut-off region extends to about 48 eV (the highest harmonic observed is the 31^st^).

**Figure 4:**
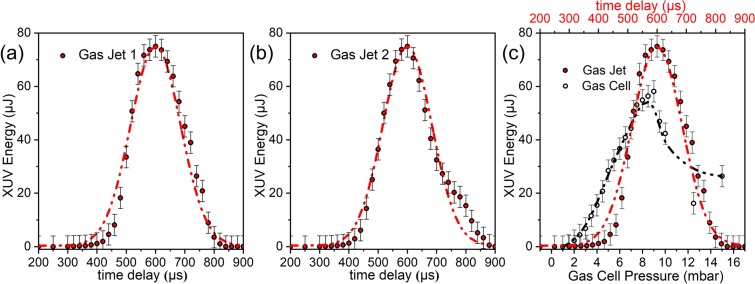
Harmonic emission using a single pulsed gas-jet and the comparison with a single gas-cell. (**a**,**b**) Pulse energy of the XUV radiation emitted by GJ_1_ and GJ_2_, respectively, as a function of the delay between the laser pulse arrival at the focus and the opening of the nozzle. Both jets are positioned at z = 0. The time delay of ≈600 μs corresponds to the value where the laser pulse meets the maximum atomic density. The dots are the measured data and the red line is a Gaussian fit. (**c**) Comparison of a single gas jet vs 10 cm long gas cell yield for optimized conditions.The upper part axis represents the time delay of the pulsed nozzle while the lower one the measured pressure of the Gas cell. In all panels the generated medium was Ar, while the XUV energy was determined by PD_XUV_ placed after an Al filter.

**Figure 5:**
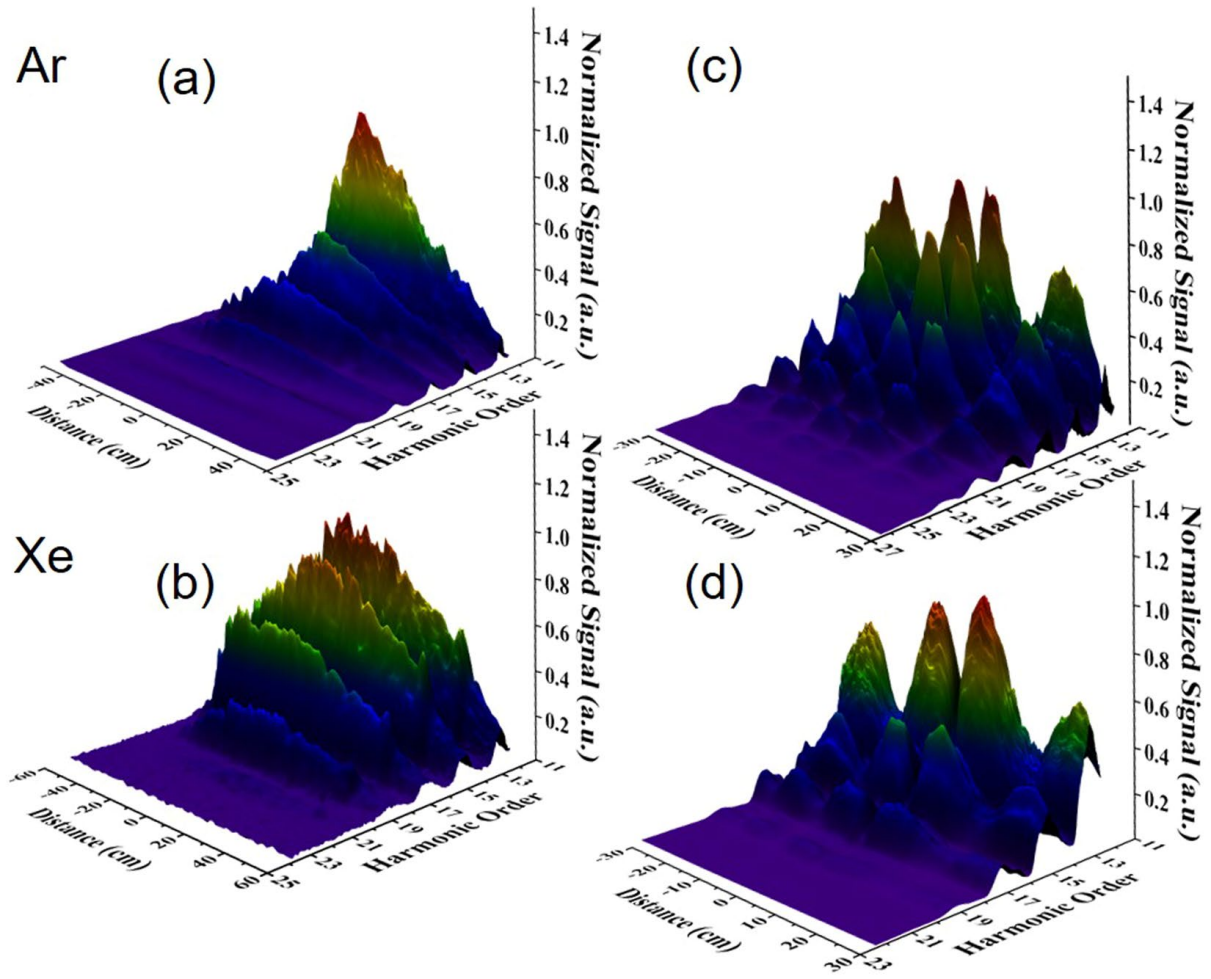
Harmonic generation in single and dual gas-jet configuration. Generation of GW high-harmonics using single (**a**,**b**) and dual gas-jet (**c**,**d**) configuration for Xe and Ar. In all panels the corresponding harmonic signal was determined by recording the single-photon photoelectron spectra produced by the interaction of Ar gas with the incoming XUV beam after passing trough the Al filter.

Figure 4 shows the dependence of the energy of the XUV radiation (integrated over the Al-filter-selected harmonics spectrum and measured with the PD_XUV_) on the argon gas pressure (changed by varying the delay of the gas nozles, both positioned at z = 0, with respect to the arrival time of the laser pulse) as well as the comparison between one gas jet and one gas cell with respect to the XUV energy emission. In particular, Fig. 4a shows the emitted XUV pulse energy as a function of the time delay between the trigger pulse of the GJ_1_ nozzle opening and the laser pulse, for an arbitrary IR intensity well below the saturation threshold. The emission maximizes for a time delay of 600 μs. At this value the harmonic signal was then further optimized by setting the IR intensity just below the ionization saturation intensity. Figure 4b shows essentially the same behavior for GJ_1_ and GJ_2_. Figure 4c is devoted to the comparison between the XUV pulse energy obtained when using a single gas jet and a cell in the present beam line. It presents the XUV pulse energy emitted by (i) GJ_1_ as a function of the pulsed nozzle time delay and (ii) by the gas cell as a function of the cell gas pressure. For the given cell length of 10 cm, the emission maximizes for a pressure value between 8 and 9 mbar. The maximum harmonic yield in the cell is found to be slightly lower (~25%) than the one of the gas jet. In these measurements Ar is used as generating medium and thus the pulse energy throughput is not the highest possible. Apart from the gas-jet/cell comparison measurement, the beam-line is operated exclusively with gas-jets, mainly because at 10 Hz repetition rate they consume less gas, and because of their demonstrated slightly higher measured XUV energy throughput. After opting for the GJ configuration as the preferable one for the beamline of this work, experimental investigations focused on maximizing the photon flux of the emitted XUV radiation. Measurements of the single GJ emission by varying the medium position relatively to the driving pulse’s focus are depicted in Fig. 5a,b for Ar and Xe respectively. The *x*-axis reveals the harmonic order, measured in the photoelectron spectrum produced by the unfocused XUV beam, the y-axis depicts the distance of gas jet from the position of the IR focus and the z-axis the XUV pulse energy. Having optimized the emission resulting from the single GJ configuration further enhancement of the harmonic yield was achieved by applying quasi- phase-matching conditions using two gas jets. The same gas is used in both jets. Results are shown in Fig. 5c,d. The dependence of the harmonic yield, generated by Ar and Xe gas, on the distance between GJ_2_ and GJ_1_ is shown in Fig. 5c,d, respectively. The x-axis denotes the distance between the two jets, the y-axis the harmonic order and the z-axis the XUV pulse enegy. GJ_1_ is positioned at fixed z ≈ 0 while GJ_2_ moves at variable positions.

All spectra emitted by Ar extend to higher cut-off energies than those emitted by Xe due to the higher ionization energy of Ar, while the pulse energy is lower than the one in Xe due to the lower conversion efficiency of Ar^61,62^. When two jets (filled with the same gas) are used a clear modulation of the signal is observed as a function of the jet distance. This is attributed to the quasi-phase matching resulting from the jet distance dependent Gouy phase and it is verified by numerical calculations^59^. The maximum measured pulse energy at the source is: I) 75 μJ (one jet) and 130 μJ (two jets), for Ar driven by 45 mJ IR pulse energy; and II) 135 μJ (one jet) and 230 μJ (two jets), for Xe driven by 25 mJ IR pulse energy. This last value corresponds to ~5·10^13^ photons/pulse, a photon flux that competes with photon fluxes of FELs in this spectral region. More details on the above quasi-phase-matching generation scheme and XUV throughputs can be found in ref. ^59^.

**Figure 6:**
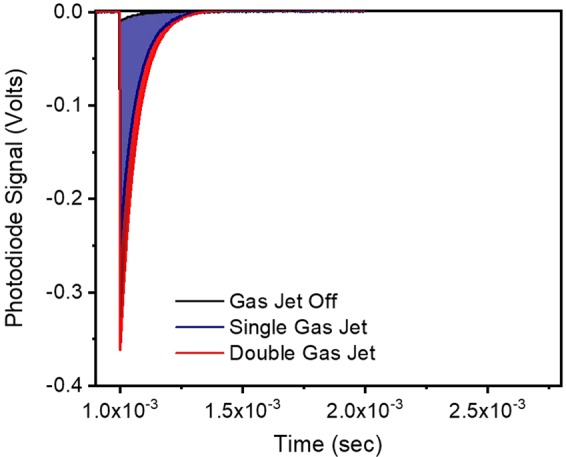
Measurement of the XUV energy. XUV photodiode signal obtained with one GJ (blue shaded area), two GJs (red shaded) and with the harmonic generation switched off (black line). For the extraction of the pulse energy the XUV photodiode quantum efficiency as a function of photon energy provided by the manufacturing company Opto Diode Corp was used.

The pulse energy measurement procedure followed is described below. Once optimization of harmonic emission is achieved, the XUV Photodiode (Opto Diode AXUV100G) is placed after the Sn filter (F). Figure 6 shows the photodiode signal of the radiation transmitted through the Sn filter produced with the single (blue shaded area) and the dual (red shaded area) GJ configuration. The black line is IR light detected by the PD_XUV_, when the gas jet of the HH generation was off. Although significantly small, this signal was subtracted from the measured total one, when harmonic generation was on.

The signal was measured with an oscilloscope (50 Ω input impedance) and the measured trace was integrated. The pulse energy *E*_*PD*_ measured at the position where the photodiode was placed is calculated by

$${E}_{PD}=\sum _{q}\frac{{n}_{e}\cdot w\cdot h{v}_{q}}{{\eta }_{q}}\cdot e$$

where *q* is the harmonic order, *n*_*e*_ is the number of produced photoelectrons, *w* is the statistical weight of the qth harmonic, *hν*_*q*_ is the harmonic photon energy, *η*_*q*_ is the photodiode quantum efficiency of the photodiode and *e* is the electron charge. The photoelectron number is given by

$${n}_{e}=\frac{{S}_{T}-{S}_{IR}}{e\cdot R}$$

where *S*_*T*_ *is* the total time integrated photodiode signal, *S*_*IR*_ *is* the time integrated photodiode signal when the harmonic generation is off, *e* is the electron charge and* R* is the oscilloscope impedance. The quantum efficiency of the photodiode as a function of the photon energy is provided by the manufacturing company (See legend of Fig. 6). The pulse energy *E* at the harmonic generation source is given by:

$$E=\sum _{q}\frac{{n}_{e}\cdot w\cdot h{v}_{q}}{{\eta }_{q}\cdot {R}_{q}^{Si}\cdot {T}_{q}^{Sn}}\cdot e$$

where ${T}_{q}^{Sn}$ is the 4% transmission of the Sn filter in this spectral region measured by recording the harmonic spectrum with and without filter, and ${R}_{q}^{Si}$ is the ~50–60% reflectivity of the Si plate. It is worth noting that after having published in ref. ^59^ the above given XUV pulse energies, a second slightly different calibration curve was published in the documents of the manufacturing company of the photodiode. Using this second calibration curve the above given and in ref. ^59^ published XUV pulse energy values reduce by 35% i.e. for Ar 48 μJ (one jet) and 85 μJ (two jets) and for Xe 88 μJ (one jet) and 150 μJ (two jets) for Xe.

### Temporal resolution

The temporal resolution of the beam line has been tested by measuring the beam pointing stability at the end station, the performance of the split mirror device and its interferometric stability. This has been done using the IR laser beam and a CW diode laser at 532 nm wavelength.

**Figure 7:**
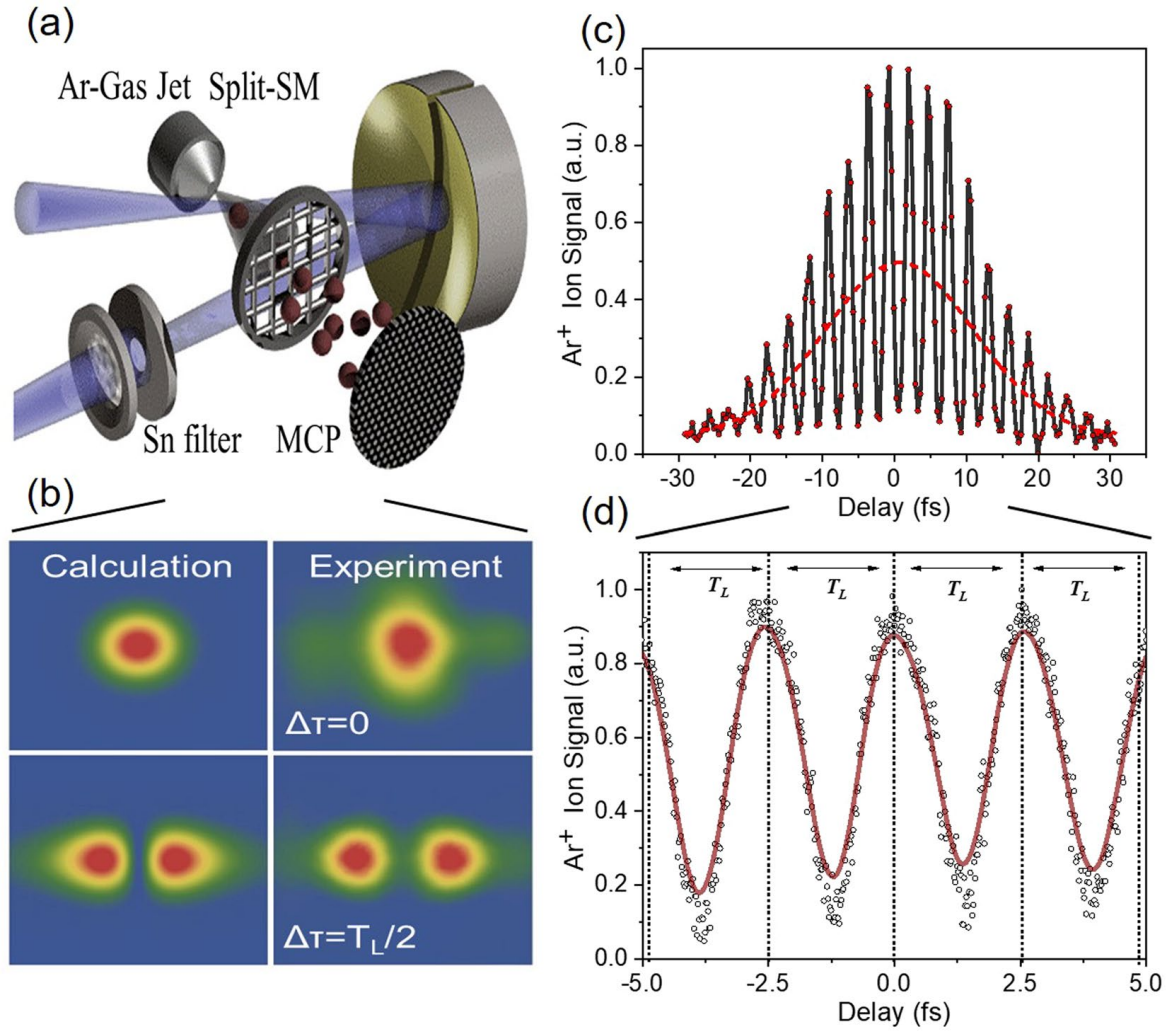
Split mirror arrangement. (**a**) A schematic of the experimental set-up of the autocorrelator consisting of a split spherical mirror and a TOF spectrometer. (**b**) Calculated (left panels) and measured (right panels) transverse intensity distribution of a CW 532 nm laser at the focus of the spherical mirror for Δτ = 0 and Δτ = *T*_*L*_/2 (double maximum distribution) delay. (**c**) High-order autocorrelation trace of the fundamental laser field (IR) obtained measuring the Ar^+^ yield as a function of the time delay between the two pulses produced by the split mirror. For this acquisition, the harmonic generation was turned off and the Sn filter was removed. (**d**) Expanded area of the AC trace. The signal is oscillating at the laser period of 2.67 fs.

Figure 7a shows a schematic of the split mirror assembly. The focal area of the gold coated spherical mirror was magnified by a lens and imaged by a CCD camera. Figure 7b shows the calculated (left panels) and measured (right panels) images of the focal spot area for two different delays, i.e. for two different displacements of the one-half of the mirror. The upper pannels show the intensity distribution at the focus formed when the phase difference between the two parts of the laser wave-front reflected by the two mirror halves is equal or close to 2*n*π, *n* = 0, 1, 2, 3…, i.e. when the delay between the two wave-fronts is ~*n*T_L_, with T_L_ being the period of the laser field.

The phase difference is controlled by finely adjusting the position of the piezoelectric translator connected with the one part of the split mirror. The position of the piezo translation stage was measured by a capacitive sensor feedback system of the piezo system. When the phase difference of the two wave-fronts becomes equal or close to (2*n* + 1)π, *n* = 0, 1, 2, 3…., i.e. when the delay between the two wave-fronts is (*n* + 1/2)T_L_, the intensity distribution at the focus splits into two bright spots shown at the lower part of Fig. 7b. The two bright spots ideally have the same intensity. Additionally, a higher-order autocorrelation trace of the fundamental laser field (IR) was recorded. For this acquisition, the harmonic generation was turned off and all filters were removed, thus ionization of Ar occurs only through the fundamental laser frequency by multi-IR-photon absorption.

The measured trace is shown in Fig. 7c where the interferometric interference fringes are clearly visible. The red dashed line is the cycle average of the data. The interference fringes are used for the calibration of the delay scale of the measured autocorrelation traces. The period of the observed oscillation, depicted in the expanded AC area trace in Fig. 7d is equal to the laser period that is 2.67 fs, where the red line is a cosine fit in the measured data.

**Figure 8:**
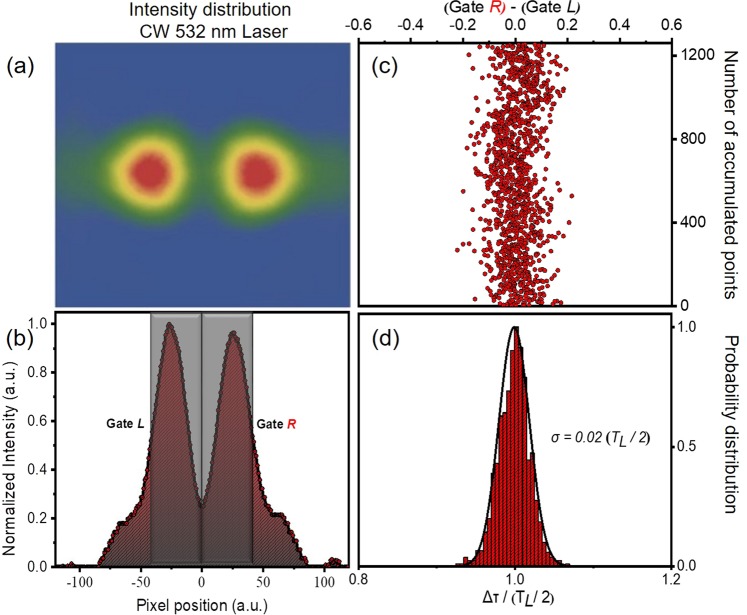
Stability measurements of the split-mirror autocorrelator. (**a**,**b**) Measured transverse intensity distribution of a CW 532 nm laser at the focus of the spherical mirror for Δτ = T_L_/2 (double maximum distribution) delay. It is noted that in this graph T_L_ corresponds to the period of the 532 nm CW laser. (**c**) The difference of the integrated signals of the Gate *L* and *R*. (**d**) Probability distribution of the above difference (1260 points were accumulated). The standard deviation of the mean yields a temporal resolution of ∼17 asec.

Shot-to-shot fluctuations of the XUV intensity distribution may be introduced because of: i) the non-perfect pointing stability of the laser and consequently of the XUV beam and ii) mechanical instabilities of the split mirror arrangement. The above factors affect the interferometric stability of the delay line. The interferometric stability of the split mirror was measured using a CW laser of λ = 532 nm by the following procedure. The displacement of the two halves of the spherical mirror was fixed such as introducing a delay of T_L_/2 (Fig. 8a). Consequently in the line-out of the focal spot area, the integrated areas of the two gates *L* and *R*, introduced in Fig. 8b, are essentially equal. Any deviation from this picture can be correlated to the instability of the split spherical mirror, since it originates from the optical path difference between the two interfering wave fronts. The interferometric stability of the split mirror device is extracted from the standard deviation of the mean value of the probability distribution for 1260 points as a function of time and is found to be ≈17 asec (Fig. 8c,d).

**Figure 9:**
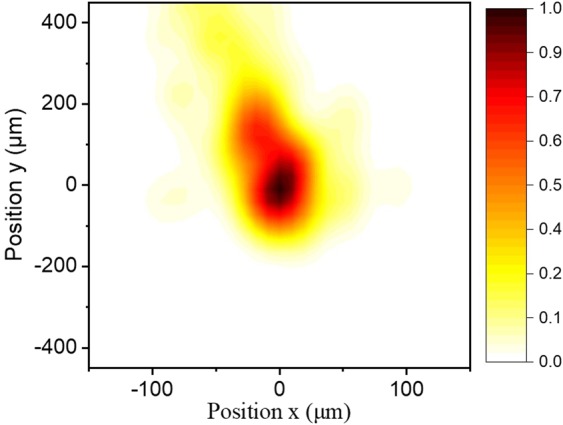
Measurement of the IR laser pointing instability. The contour illustrates the shot to shot distribution of the maximum of the IR laser intensity distribution measured just before the split mirror. The colorbar shows the normalized probability distribution of laser shots.

The interferometric stability of the device may be different when the IR laser is used as its pointing stability is not the same with that of the CW one. The pointing stability of the IR was measured with an IR beam profiler placed just in front of the split mirror. The shot to shot position of the maximum of the intensity distribution is plotted in Fig. 9 for 150 laser shots. The mean FWHM of the contour is about 75 μm and thus substantially smaller than the 3 mm FWHM of the XUV intensity distribution at the split mirror, not affecting the measured interferometric stability and time resolution.

## Non-Linear XUV Optics Using the 20-GW XUV Beam-Line

The highest focused XUV intensity achieved was ~7·10^15^ W/cm^2^ assuming a 10 fs long pulse train envelope^59^, an XUV focal spot size of 2 μm measured with an ion-microscope^67^, the gold reflectivity (~12%) of the spherical mirror, the ~60% reflectivity of the Si plate, the ~20% transmition of the Sn filter for the given wavelengths and the 230 μJ generated pulse energy at the harmonic source. Such intensity allows the investigation of multi-XUV-photon multiple ionization. Here, we summarize the results obtained in He, Ar and Ne atoms. Some of the multi-XUV-photon schemes of this chapter have been used for the measurement of the attosecond pulse duration through 2^nd^ IVAC measurements (schemes in He and Ar), which will be described in the next section.

**Figure 10:**
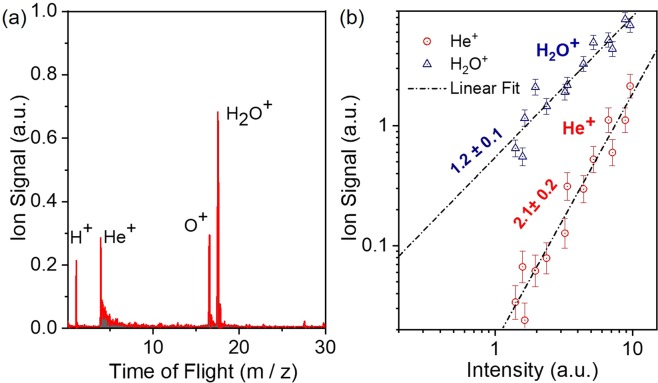
2-XUV-photon ionization process of He. (**a**) TOF mass spectrum produced by the interaction of the XUV comb (11^th^−15^th^) with He gas.(**b**) XUV intensity dependence of the He^+^. The slope of 2.1 ± 0.2 ascertains the second-order nonlinearity of the ionization process. The intensity axis in (b) has been calibrated using the O^+^ ion signal, which is linear with the XUV intensity.

**Figure 11:**
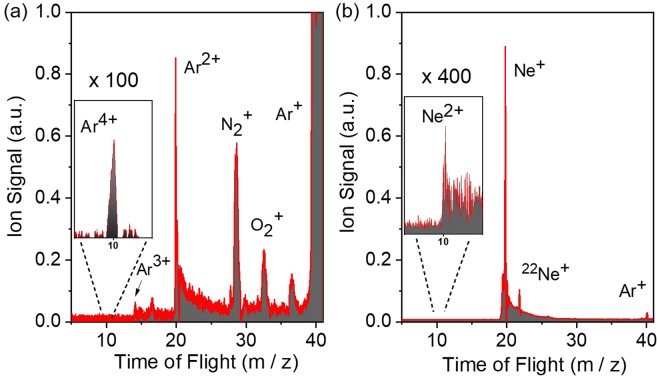
Time of Flight mass spectrum of Ar and Ne. (**a**) TOF mass spectrum produced by the interaction of the focused 11^th^−15^th^ harmonics with Ar. The spectrum shows multiple charged Ar ions (Ar^*n*+^) with *n* up to +4. (**b**) Measured Ne ion mass spectrum produced by the XUV radiation. In the spectrum two Ne^+^ ion mass peaks are to be seen corresponding to the two most abundant isotopes, ^20^Ne and ^22^Ne. A small Ne^2+^ peak and an Ar^+^ peak are also observed. The Ar^+^ peak originating from residual Ar gas is used for calibration of the mass ion spectrometer.

**Figure 12:**
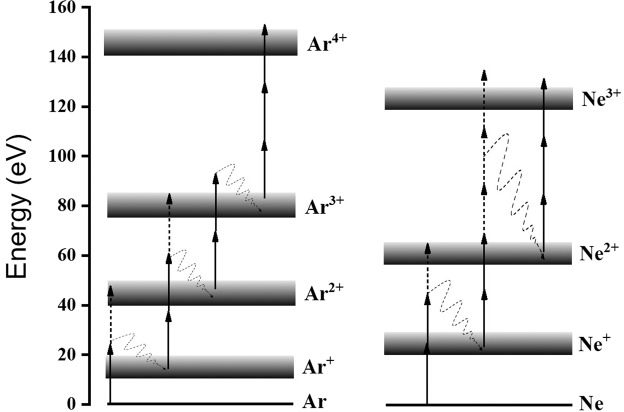
Multi-XUV-photon multiple ionization scheme. The ionization energy level schemes for Ar and Ne (excluding higher order processes (ATI) and decays to excited ionic states) depicting the direct and sequential channels.

**Figure 13:**
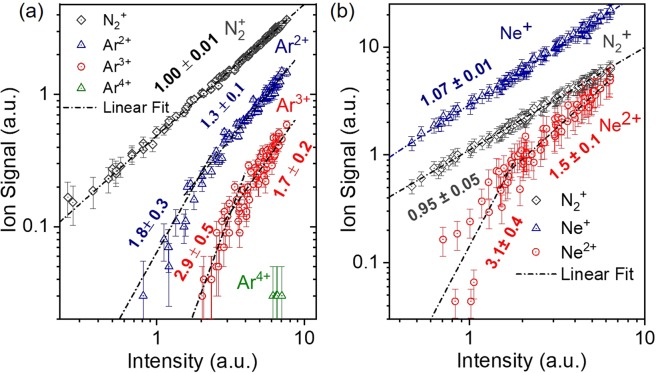
Ion yield dependence on the XUV radiation. XUV intensity dependence of different charge states of Ar (**a**) and Ne (**b**). In both panels the black dashed lines depict a linear fit on the raw data and the error bars represent one standard deviation of the mean. The slopes of the lines in both measurements, are in agreement with lowest-order pertubation theory i.e. with the order of the underlying non-linear process.

Figure 10a shows the measured ion mass spectrum of He, in which He^+^ is clearly observable. It should be noted that for this measurement an Sn filter was used. The XUV intensity dependence of the ion yield is depicted in Fig. 10b. The slope of the fitted line in the He^+^ data is 2.1 ± 0.2, as expected for the underlying two photon ionization process, while the slope of the line fitted in the H_2_O + date is 1.2 ± 0.1, as water molecules are single photon ionized at the XUV photon energies used. The verified two-XUV-photon ionization of He is a very convenient process in performing 2^nd^ order autocorrelation measurements of XUV radiation with wavelengths >51 nm. The Ar and Ne ion TOF mass spectra are shown in Fig. 11a,b respectively. The latter reveals the formation of singly and doubly charged Ne, while the former shows recorded charge states of Ar up to +4 (Ar^4+^). Figure 12 depicts the ionization schemes of Ne and Ar, while Fig. 13a shows the dependence of the Ar^2+^, Ar^3+^ and N_2_ ^+ ^yield and Fig. 13b the dependence of the Ne^+^, Ne^2+^ and N_2_^+^ yield on the XUV pulse intensity *I*_XUV_.

The Ar^+^ ion mass peak of Fig. 11b was used for the calibration of the XUV energy scale (x-axis) of the Ne ion yield power dependence graph (Fig. 13b). The black dashed-dot lines in Fig. 13 are linear fits to the raw data. The error bars represent one standard deviation of the mean.

The results for Ar gas have been extensively discussed in ref. ^59^. In brief, intensity dependence measurements performed for Ar^+^, Ar^2+^ and Ar^3+^ were supported by numerical calculations revealing the dominant channels of these multi-XUV-photon multiple ionization studies. Comparison with the data obtained using FEL source indicates that there are differences in multiphoton ionization induced by the two different sources, which can be attributed to the different photon statistics of the two sources^59^.

As expected for a single-photon ionization process, the dependence of the Ne^+^ and N_2_^+^ yields on *I*_XUV_ is linear (Fig. 13b). The slope of the Ne^2+^ yield is found to be 3.1 ± 0.4 compatible with a three-photon ionization process. For the photon energies employed in this experiment both the sequential and the direct double ionization of Neon are three photon processes. Above the ionization saturation intensity the slope becomes 1.5±0.1. For the next charge state, i.e. Ne^3+^, six or more photon absorption is required. This charge state is not observable in the measured ion mass spectra.

## Temporal Characterization of the Attosecond Pulse Trains

After having set up, characterized and tested the high photon flux beam-line, measurements towards temporal characterization of the APTs synthesized by the harmonic spectrum have been performed. It is worth noting that in these measurements the diameter of the aperture (A) in the “XUV filtering and diagnostics” chamber (Fig. 1) was reduced as to decrease the XUV signal to about half of its maximum value. Thus the outer part of the XUV beam cross-section was blocked. Consequently i) aberrations in the XUV beam were reduced and ii) the ratio of the short to long trajectory contribution in the transmitted XUV beam was increased. The method used is the 2^nd^ IVAC utilizing the delay line and TOF spectrometer discussed in section 3 and shown in Fig. 7a. As second order non-linear process, the two-XUV-photon ionization of both Ar^+^ and He were used. This is in order to demonstrate different two-photon schemes that can be used in pulse duration measurements at higher photon energies. In performing the 2^nd^ IVAC measurements the gas pressure in the interaction area was kept as low as possible in order to minimize the space charge effects which become visible by broadening the TOF ion-mass peaks. In Ar the traces are obtained by the superposition of the harmonics transmitted through the Sn filter. Before saturation, the Ar^2+^ yield as a function of the *I*_XUV_ in log-log scale has a linear dependence with slope ~2^59^. This slope is compatible with either two-XUV-photon direct double ionization of Ar or two-XUV-photon ionization of Ar^+^ after saturation of the single photon ionization of Ar (see Fig. 12). Numerical calculations in^59^ have shown that the latter channel, i.e. saturated single photon ionization of Ar followed by two photon ionization of Ar^+^ is the dominant channel in the *I*_XUV_ range in which the present experiments have been performed.

**Figure 14:**
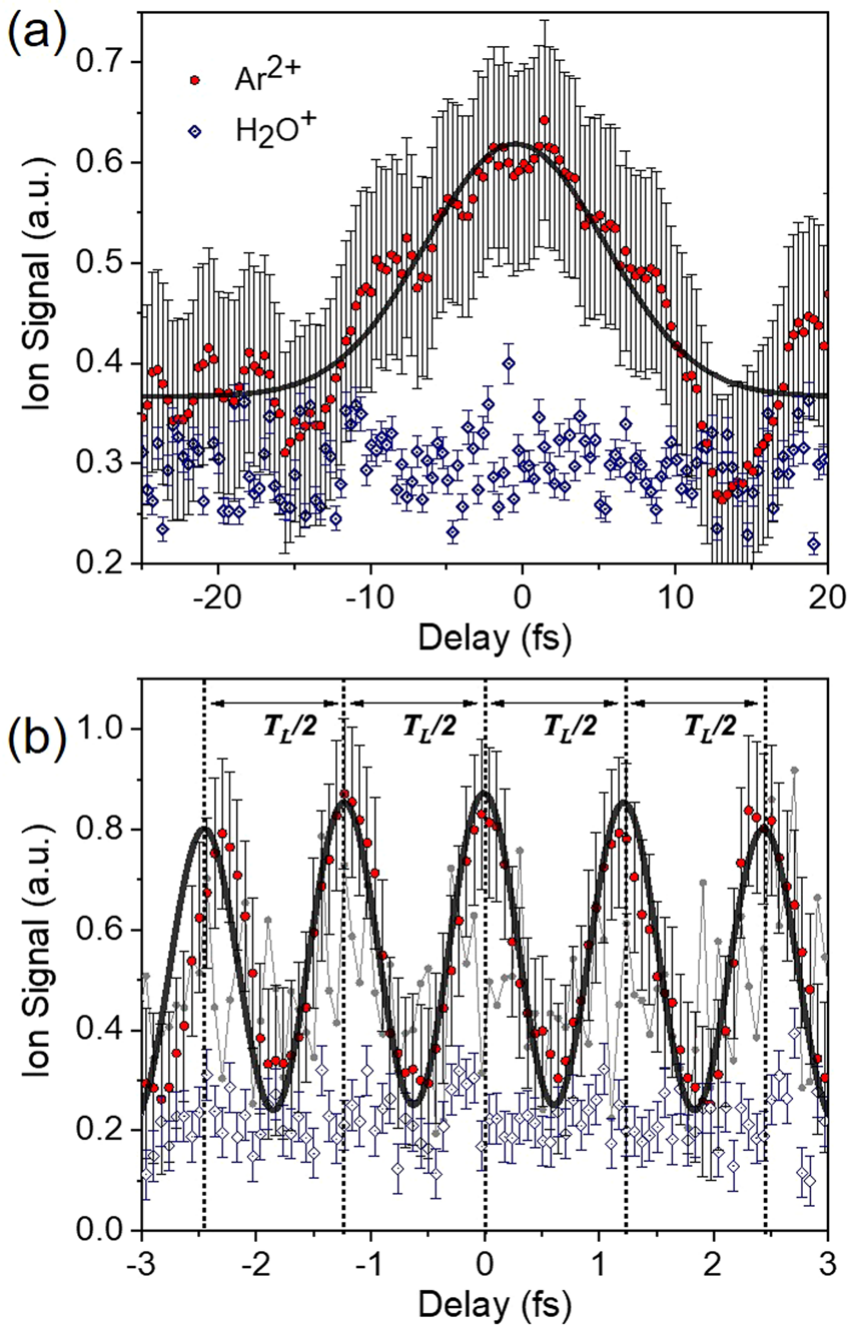
Measured 2^nd^ IVAC trace i.e. Ar^2+^ ion signal as a function of the XUV-XUV delay line. The XUV radiation is produced by a single gas jet of xenon and is transmitted through a Sn filter. (**a**) A coarse time delay scan with 350 asec step is revealing a modulation in Ar^2+^ ion signal represented by the red circles, while the blue rhombus depicting the single photon ionization of H_2_O shows no modulation. A Gaussian fit in the data points of Ar^2+^ yields a time duration of τ_XUV_ = 9.8 ± 0.9 fs. (**b**) A fine scan with time delay step of 50 asec. The gray circles correspond to the raw data recorded for Ar^2+^. The moving averages of the raw data taken over 10 points are represented by the red circles. The black curve is a fit of a sequence of gaussian pulses in the averaged points.

Measured 2^nd^ IVAC traces are shown in Fig. 14a,b. The blue rhombus is the trace obtained from the single photon ionization of H_2_O. As expected for a linear process the IVAC shows no modulation. The Ar^2+^ ion yield (produced by the XUV radiation generated using only one gas jet) is measured here as a function of the delay between the two XUV pulses introduced by the translation of one part of the bisected spherical mirror. The gas jet in the HHG chamber was set at 20 cm after the laser focus in order to minimize the contribution of the long electron trajectory. Low temporal resolution scans recorded with a step of 350 asec have been performed in determining the duration of the APT envelope (Fig. 14a). The red points are the raw data, averages of 50 laser points and the error bar corresponds to the standard deviation of the mean value. The black curve is a Gaussian fit to the data. The fit results in an XUV pulse envelope having a duration of 9.8 ± 0.9 fs, verifying the estimated duration used in ref. ^59^. A fine scan using a time delay step of 50 asec is shown in Fig. 14b. The Ar^2+^ ion yield, as expected, is modulated with the half period of the driving field. The gray circles are the recorded raw data (averages of 50 laser shots). The raw data in the fine scan of Fig. 14b are fluctuating around the mean value mainly due to interferometric instabilities (within the cycle of the XUV field) and XUV beam pointing instabilities, which are both enhanced by the non-linearity of the process. Long averaging and calculating moving averages substantially reduce the strong shot-to-shot fluctuation of the recoded data. The red circles are the moving averages of the raw data taken over 10 points. The black curve is fit of a series of Gaussian distributions to the averaged points. In this fit the free parameters are the common width, height of the Gaussians as well as the peak to peak distance. Furthermore the comb of Gaussians are multiplied by a fixed envelope distribution taken from the fit of Fig. 14a. The pulse width resulting from the Gaussian distributions is found to be τ_XUV_ = 650 ± 80 asec. The error of 80 asec appearing in all measurements is the largest resulted standard deviation, among all the fits in the raw data of all measured traces. The above pulse duration of the attosecond pulses in the APT is synthesized essentially by the three harmonics 11^th^, 13^th^ and 15^th^. Since here only one gas jet was utilized, the APT beam-line power to be rigorously reported is 11.0 ± 3.5 GW, the error originating mainly from the uncertainty in the calibration of the XUV photodiode.

**Figure 15:**
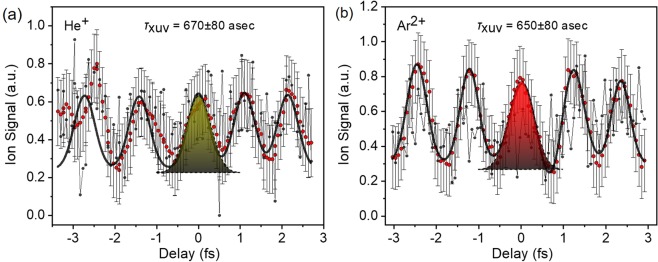
(**a**) Measured 2^nd^ IVAC trace of the He^+^ (**b**) 2^nd^ IVAC trace of the Ar^2^^+^ ion signal as a fuction of the delay of the XUV-XUV delay line. The gray circles correspond to the raw data recorded, the moving averages of the raw data taken over 10 points are represented by the red circles. The black curves is a Gaussian fit over the averaged points. The temporal width of the Gaussian fit corresponds to a pulse duration of 670 ± 80 as and 650 ± 80, for He^+^ and Ar^2+^ respectively.

The two-XUV-photon ionization of He^+^ has also been used to measure the produced APTs through 2^nd^ IVAC, shown in Fig. 15a and alongside with Fig. 15b showing a 2^nd^ IVAC trace of Ar^2+^. The trace of Fig. 15b is a different run than the one shown in Fig. 14b verifying reproducibility of the results. All points, error bars and curves are as those in Fig. 14b. with the only difference being that here we do not use any envelope distribution in the fit either for the He or the Ar trace. This is because in these runs the peak height distribution within the error bars did not depict any envelope type modulation. The pulse duration measured using He as non-linear medium is 670 ± 80 asec and the one of the superposition of harmonics 11^th^, 13^th^ and 15^th^ measured in Ar^2+^ is the same as the one of Fig. 14b. The two values are well within the error bar and thus essentially identical.

The measured durations here are similar to those retrieved in previous experiments implemented in a 3 m focal length beam-line applying 2^nd^ IVAC in two photon ionization of He but about 65% longer than those measured through the RABBIT technique^39^. The discrepancy between the 2^nd^ IVAC and RABBIT originates from the fact that 2^nd^ IVAC measures averages of spatiotemporally dependent pulse durations and the contribution of both long and short trajectories, while RABBIT measures average phases^39^. An additional effect to be considered is pointed out recently in refs. ^66,68^. Different harmonics, due to their different divergence are focused at different positions, have different focal spots and therefore lead to only partial spatial overlap of the superimposed harmonics and to different Gouy phase contributions in the harmonic superposition. At specific conditions, e.g. spectrum with harmonics of very different order, the spatial overlap becomes notably small and the Gouy phase difference large, thus reducing a lot the temporal confinement. 2^nd^ IVAC is sensitive to these effects and thus reveals fairly realistic pulse durations. However, for the three harmonics employed in this experiment substantial spatial overlap is present as indicated by the results of a recent work^69^, were separation of the harmonic foci was not observed.

In order to verify the significant spatial overlap of the three harmonics used in the 2^nd^ IVAC measurements we have performed calculations of the focal areas of the three harmonics, for a bandwidth spanning from the 9^th^ to the 17^th^ harmonic and Xe gas as harmonic generating medium. We are using the expression ${\theta }_{S,L}=\frac{{\lambda }_{q}}{\pi {w}_{q}}\sqrt{1+4{\alpha }_{S,L}^{2}{I}_{L}^{2}\frac{{w}_{q}^{4}}{{w}_{f}^{4}}}$ of the divergence of the harmonics originating from the electron short (*S*) and long (*L*) trajectories at the point of the interaction for the harmonic order *q*, as given in ref. ^70^. ${\lambda }_{q}$, ${w}_{q}$, are the wavelength and the beam waist of the harmonic *q*, ${w}_{f}$ is the waist size of the laser beam, ${\alpha }_{S,L}$ is the *S* and *L* trajectory coefficient and *I*_*L*_ is the IR driving laser peak intensity. The beam waist was measured at the emission plane and it was found to be *w*_*f*_ ≈ 350 μm. Using Gaussian optics the harmonic beam waist can be obtained by ${w}_{q}=\frac{{w}_{f}}{\sqrt{{q}_{eff}}}$ where *q*_*eff*_ is the effective nonlinearity coefficient with *q*_*eff*_ ≈ 5 for all the harmonics laying in the plateau of the harmonic spectrum^69,71,72^. For a peak intensity 10^14^ W/cm^2^ all the studied harmonics are laying in the plateau and the trajectory coefficient *α*_*S,L*_ is extracted by solving the three-step semi-classical model^73^. The results are summarized in Table 1.

**Table 1:** Parameters of the 9^th^ to the 17^th^ harmonics generated in Xe.

Harmonic order	9	11	13	15	17
*λ*_*q*_(nm)	88.9	72.7	61.5	53.3	47.0
*α*_*S*_	1	0.435	−0.4	−1.5	−2.96
*α*_*L*_	−23.97	−23.56	−22.88	−21.96	−20.71
*θ*_*S*_(mrad)	0.195	0.15	0.127	0.126	0.148
*θ*_*L*_(mrad)	1.74	1.40	1.15	0.96	0.79

The intensity of the laser field used is 10^14^ W/cm^2^ and the unit for *α* is 10^−14^ W^−1^·cm^2^.

**Figure 16:**
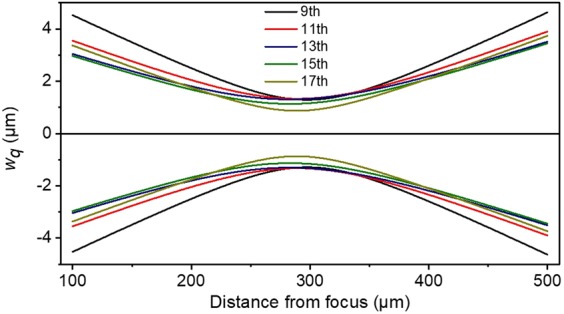
Calculated focal areas of the five harmonics 9^th^, 11^th^, 13^th^, 15^th^ and 17^th^. The graph depicts the harmonic beam waist as a function of the distance from the focus of the spherical mirror. The x-axis shows the focus position of the harmonics on the propagation axis. The focus position of the harmonics is found to be ≈ 290 μm away from the focus of the 5 cm mirror. Their beam waist ratios are 0.99:1.01:1:0.87:0.67 for harmonic 9^th^, 11^th^, 13^th^, 15^th^ and 17^th^ respectively.

After extracting the divergence of the different harmonics, the virtual source positions for each of the generated harmonics is calculated assuming only short trajectory contribution. The focus positions of the harmonics, after reflection on the spherical mirror of focal length *f* = 5 cm, are calculated using geometrical optics. Here the paraxial approximation is applied since the divergence of the harmonics is below the paraxial limit. The results of the calculations are shown in Fig. 16.

**Figure 17:**
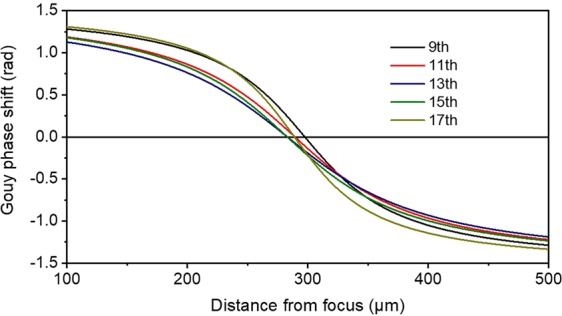
Calculated Gouy phase shift of the five harmonics 9^th^, 11^th^, 13^th^, 15^th^ and 17^th^. The x-axis shows the focus position of the harmonics on the propagation axis. The focus position of the harmonics is found to be ≈ 290 μm away from the focus position of the 5 cm mirror.

The distance of the positions of the five foci is 8.2 μm between the 9^th^ and 11^th^, 6.3 μm between the 11^th^ and 13^th^ harmonic, ~0 μm between the 13^th^ and 15^th^ harmonic, 5.9 μm between the 15^th^ and 17^th^ harmonic and thus it is negligibly small with respect to their confocal parameter (≈170 μm for 13^th^ harmonic). The size of the focal spots is slightly different. The ratios for the beam waists at the focus are 0.99:1.01:1:0.87:0.67 for the harmonics 9^th^, 11^th^, 13^th^, 15^th^ and 17^th^ respectively. Under these conditions, the spatial overlap of the five harmonics is substantial. The Gouy phase at the beam waist for each harmonic can be calculated and is shown in Fig. 17. Its variation for the different harmonics (assuming as central frequency the 13^th^ harmonic) ${\varphi }_{9}=0.24\,{\rm{rad}},\,{\varphi }_{11}=0.08\,{\rm{rad}},\,{\varphi }_{13}=0\,{\rm{rad}},\,{\varphi }_{15}=0\,{\rm{rad}},\,{\varphi }_{17}=0.13\,{\rm{rad}}$ is also negligibly small. In this case, the duration of the APT pulses is not significantly affected. In fact, the different beam waists of the 11^th^, 13^th^, 15^th^ harmonics lead to more similar amplitudes of the interfering harmonics than those generated.

Similar calculations have been further performed for the long electron trajectories, which present larger harmonic divergence. In this case, the virtual foci are placed closer to the focusing element. It is found that the difference of the foci positions between *L* and *S* trajectories is ≈30 μm which is consistent with previous experimental findings^69,72,74^ and also smaller than the confocal parameter, thus not substantially affecting the APT pulse duration in particular because the long trajectory contributions are reduced through the geometry used.

## Conclusions

In summary, a detailed description of an ultra-intense attosecond XUV beam line has been presented. A ten GW class average peak power attosecond source in the XUV spectral region 15–25 eV is demonstrated. These specifications are to our knowledge unique for an XUV source. While in a previous publication the high power of the source (20 GW) was reported^59^ attosecond confinement, although expected, could not be rigorously claimed as the previous work did not include any pulse duration measurements. In the present work, APT durations of the order of 650 asec have been measured opening the way to ten-GW class attosecond XUV sources. The source is based on harmonic generation in long (9 m) focusing geometry of the driving IR laser radiation. The pulse duration of both the APT pulses and the envelope have been measured though 2^nd^ IVAC i) in He employing two-XUV-photon ionization as a second order process as well as ii) in Ar exploiting two-XUV-photon ionization of Ar^+^ under saturation of neutral Ar ionization. Measurements with both gases resulted in the same pulse durations within the experimental error. High non-linear XUV-optics in terms of multiple multi-XUV-photon ionization of He, Ar and Ne atoms, have been further demonstrated using the above beam line. The combination of high pulse energy and short duration opens up excellent perspectives for sub-fs XUV-pump-XUV-probe experiments in all states of matter. At the same time the XUV intensity levels reached enable the study of strong field effects in the XUV spectral region. As a further perspective, scaling previous parameters of isolated attosecond pulses^64^, our source holds promise of generating few μJ level isolated attosecond pulses through polarization gating approaches. Those are advanced perspectives for the Hellenic National Research Infrastructure HELLAS-CH, part of which is the present beam line.

The results of the present work further hints towards an unprecedented performance of the two 1-kHz repetition rate attosecond beam lines of the Extreme Light Infrastructure – Attosecond Light Pulse Source (ELI-ALPS) facility currently being under implementation^75^, driven by shorter laser pulses with similar pulse energies. The geometry of one of the two beam-lines of ELI-ALPS is very close to that of the present source, while the second one is several times longer and offers phase matching control capacities. Thus, it is expected to further scale up the source throughput. The 1 kHz repetition rate of these sources in combination with the CEP stabilized driving laser will provide the by far best ever conditions for attosecond XUV-pump-XUV-probe investigations using isolated attosecond pulses and kinematically nearly complete experiments through e-e, e-ion and ion-ion coincidence measurements.
